# Expression and significance of hypoxia-inducible factor-1α and MDR1/P-glycoprotein in laryngeal carcinoma tissue and hypoxic Hep-2 cells

**DOI:** 10.3892/ol.2013.1321

**Published:** 2013-04-29

**Authors:** JIN XIE, DA-WEI LI, XIN-WEI CHEN, FEI WANG, PIN DONG

**Affiliations:** 1 Department of Otolaryngology-Head and Neck Surgery, Shanghai Jiao Tong University Affiliated First People’s Hospital, Shanghai 200080;; 2 Department of Otolaryngology-Head and Neck Surgery, Affiliated Eye and ENT Hospital of Fudan University, Shanghai 200031, P.R. China

**Keywords:** hypoxia, hypoxia inducible factor-1α, multidrug resistance, p-glycoprotein, laryngeal neoplasms

## Abstract

The present study aimed to evaluate the expression of hypoxia-inducible factor-1α (HIF-1α) and MDR1/P-glycoprotein (P-gp) in human laryngeal squamous cell carcinoma (LSCC) tissues, and also to investigate the regulation of *MDR1* gene expression by HIF-1α in Hep-2 cells under hypoxic conditions. The expression of HIF-1α and MDR1/P-gp in human LSCC tissues was examined using immunohistochemistry. The *HIF-1α* and *MDR1* gene expression in the Hep-2 cells was detected using real-time quantitative reverse transcription (QRT)-PCR and western blot analysis under normoxic and hypoxic conditions. In hypoxia, HIF-1α expression was inhibited by RNA interference. HIF-1α and MDR1/P-gp expression was high in the LSCC tissues and was associated with the clinical stage and lymph node metastasis (P<0.05). HIF-1α expression was positively correlated with MDR1/P-gp expression (P<0.01). In the Hep-2 cells, HIF-1α and MDR1/P-gp expression significantly increased in response to hypoxia. The inhibition of HIF-1α expression synergistically downregulated the expression of the *MDR1* gene in hypoxic Hep-2 cells. HIF-1α expression is positively correlated with MDR1/P-gp expression in LSCC, and the two proteins may be able to serve as potential biomarkers for predicting the malignant progression and metastasis of LSCC. HIF-1α may be critical for the upregulation of *MDR1* gene expression induced by hypoxia in Hep-2 cells.

## Introduction

It is generally known that the majority of human solid tumors contain hypoxic regions. Due to the combination of the uncontrolled, rapid growth of tumor cells and the inability of the local vasculature to supply sufficient nutrients and oxygen, hypoxia has become a significant characteristic of human solid tumors. There has been a general consensus towards the theory that hypoxia plays a central role in tumor progression and resistance to chemotherapy (1).

Hypoxia is known to upregulate the expression of hypoxia-inducible factor-1 (HIF-1), which is a basic helix-loop-helix transcription factor that plays an essential role in regulating the transcription of various target genes in response to hypoxia (2). HIF-1 is a heterodimer composed of α and β subunits. HIF-1β is considered to be an aryl hydrocarbon nuclear translocator (ARNT), whereas HIF-1α has been recognized as an oxygen-regulated subunit that mediates the function of HIF-1 (3). A series of studies have previously shown that the overexpression of HIF-1α was involved in the pathogenesis of tumor angiogenesis (4,5), invasion (6,7), metastasis (6–8) and resistance to chemotherapy (9,10). Several studies have focused on whether HIF-1α expression in human laryngeal carcinoma tissue is associated with tumor progression and lymph node metastasis. However, those conclusions have been inconsistent thus far (11,12).

It is well known that multidrug resistance (MDR) is one of the major obstacles in the treatment of cancer using chemotherapy. In human tumors, the classic MDR phenotype is conferred by the expression of MDR1/P-glycoprotein (P-gp), which is a member of the adenosine triphosphate (ATP)-binding cassette (ABC)-type transporter family (10,13). MDR1/P-gp functions as an energy-dependent membrane efflux pump that is able to regulate the intracellular drug concentrations to determine the drug sensitivity situation of the cells. MDR1/P-gp has previously been identified to play a key role in the regulation of chemosensitivity in laryngeal cancer cells (14,15). Furthermore, the downregulation of MDR1/P-gp expression has been demonstrated to be an effective way to enhance the chemosensitivity of laryngeal cancer cells to conventional chemotherapeutic agents (15). To the best of our knowledge, a series of studies have indicated that HIF-1α may take part in the regulation of *MDR1* gene expression in multiple human tumors, including colonic (16) and hepatocellular (17) carcinoma. As yet, studies have not demonstrated a correlation between the HIF-1α protein and *MDR1* gene expression in human laryngeal cancer.

The present study explored the expression and correlation of HIF-1α and MDR1/P-gp in human laryngeal squamous cell carcinoma (LSCC) tissues. The study also determined whether hypoxia exhibited an effect on the regulation of *MDR1* gene expression in Hep-2 cells, and the role of HIF-1α in the transcriptional regulation of *MDR1* gene expression in hypoxic Hep-2 cells.

## Materials and methods

### Patients and tissue samples

Paraffin-embedded surgical tissue specimens were obtained from the pathological files of 86 patients with LSCC that had been admitted to the Shanghai Jiao Tong University Affiliated First People’s Hospital (Shanghai, China). All the patients involved in the study were treated between January 1997 and December 2008, and underwent surgery for LSCC in the Department of Otolaryngology-Head and Neck Surgery. A total of 81 male and 5 female patients, with ages ranging between 37 and 84 years (median age, 51 years) were selected. None of the patients had undergone treatment prior to surgery. All patients had a histopathological diagnosis of squamous cell carcinoma, as determined by pathologists. The clinicopathological data are shown in Table I. According to the anatomical site of the primary tumor, 18 cases of supraglottic laryngeal carcinoma, 66 cases of glottic carcinoma and two cases of subglottic carcinoma were diagnosed. The disease stage was determined according to the 2002 TNM classification of the International Union Against Cancer (UICC, Geneva, Switzerland) (18). The histological grade of the tumor was determined according to the degree of differentiation (Broders’ classification). Approval for the study was obtained from the Ethics Committee of The Shanghai Jiao Tong University Affiliated First People’s Hospital and informed consent was obtained from all participants.

### Immunohistochemistry

The paraffin-embedded tissues were cut into 5-*μ*m sections, deparaffinized in xylene and dehydrated through a graded series of ethanol solutions. The antigen retrieval was performed by heating the sections for 18 min in a microwave oven with a citrate buffer (10 mmol/l, pH 6.0). The slides were washed in phosphate-buffered saline (PBS) and the endogenous peroxidase activity was halted (3% hydrogen peroxide in methanol for 10 min), followed by incubation in 10% normal goat serum for 30 min to minimize undesirable non-specific staining. The tissue sections were then incubated with the following primary antibodies-overnight at 4°C: mouse anti-HIF-1α monoclonal antibody (Millipore Corporation, Billerica, MA, USA) at 1:100 dilution and mouse anti-MDR1/P-gp monoclonal antibody (ab3366; Abcam, Cambridge, UK) at 1:40 dilution. Immunodetection was performed by a two-step immunohistochemistry procedure using the Envision System, with diaminobenzidine chromogen as a substrate (DAKO, Carpinteria, CA, USA) and according to the manufacturer’s instructions. Finally, the nuclei were counterstained using hematoxylin solution and the negative controls were incubated with PBS in place of the primary antibody.

### Evaluation of staining

The results of the immunoreactivity procedure were evaluated independently by two pathologists who had no prior knowledge of the clinicopathological patient data. The immunohistochemical assessment of the expression of HIF-1α (19) and MDR1/P-gp (20) in the human LSCC tissues was performed using a semi-quantitative scoring system, as described in previous studies. HIF-1α immunostaining was evaluated as a percentage of the nuclear positivity by counting the positive tumor nuclei, regardless of cytoplasmic staining. A positive classification was awarded if the percentage of positive tumor cells was >10%. All the other cases were considered to be in the negative category. The MDR1/P-gp immunoreactivity specimens that scored 0 were defined as in the negative category and all others were placed into the positive category.

### Cell line and culture

The Hep-2 human laryngeal carcinoma cell line was obtained from the American Type Culture Collection (ATCC, Manassas, VA, USA) and was cultured in high-glucose Dulbecco’s modified Eagle’s medium (DMEM; Gibco Corporation, Carlsbad, CA, USA) with 10% fetal bovine serum (Hyclone, Logan, UT, USA) and antibiotics (100 IU/ml penicillin and 100 IU/ml streptomycin). The normoxic control cells were incubated in a humidified atmosphere consisting of 95% air and 5% CO_2_ at 37°C. For the hypoxic conditions, the well-developed Hep-2 cells were cultured for 24 h in a modulator incubator chamber at 37^o^C, with 94% N_2_, 1% O_2_ and 5% CO_2_.

### Inhibition of HIF-1α expression in Hep-2 cells by RNA interference

A double stranded siRNA oligonucleotide targeting the *HIF-1α* gene (sense, 5-CUGAUGACCAGCAACUUGAdTdT-3 and anti-sense, 5-UCAAGUUGCUGGUCAUCAGdTdT-3) was synthesized according to the human *HIF-1α* complementary deoxyribonucleic acid (cDNA) sequence in the gene bank (NM001530; Shanghai Genepharma Co., Ltd., Shanghai, China), which was previously identified (21). A non-specific control siRNA (forward, 5-AGUUCAACGACCAGUAGUCdTdT-3 and reverse, 5-GACUACUGGUCGUUGAdTdT-3) was designed by a basic alignment search tool (BLAST) search (National Center for Biotechnology Information database, Bethesda, MD, USA) and synthesized by the Genepharma Company. This was not homologous to any human transcripts in the records. The Hep-2 cells were incubated in an antibiotic-free medium for 24 h prior to transfection with siRNA (100 nM) using Lipofectamine 2000 (Invitrogen, Carlsbad, CA, USA) according to the manufacturer’s instructions. The cells were harvested and examined following 24 h of transfection.

### Real-time quantitative reverse transcription (QRT)-PCR for HIF-1α and MDR1

The total RNA was extracted by Trizol reagent (Invitrogen) according to the manufacturer’s instructions. The RT reaction was performed using the ExScriptRT reagent kit (Takara, Shiga, Japan) in a final reaction mixture consisting of 1 *μ*g total RNA, 4 *μ*l 5X ExScript buffer, 1 *μ*l OligodT primer, l *μ*l deoxynucleotide triphosphate (DNTP) mixture, 0.5 *μ*l RNase inhibitor, 0.5 *μ*l ExScript RTase and RNase-free water to 20 *μ*l liquid. The RT reaction comprised of an initial step at 42°C for 15 min and was terminated by heating at 95°C for 2 min. The QRT-PCR was then executed using the ABI Prism 7900 real-time PCR system with SYBR-Green 1 (Invitrogen). The primers were as follows: *HIF-1α* forward, 5′-AACATAAAGTCTGCAACATGGAAG-3′ and reverse, 5′-AACATAAAGTCTGCAACATGGAAG-3′; *MDR1* forward, 5′-CTTCAGGGTTTCACATTTGGC-3′ and reverse, 5′-GGTAGTCAATGCTCCAGTGG-′3; *GAPDH* (internal control) forward, 5′-CATCTTCCAGGAGCGAGA-′3 and reverse, 5′-TGTTGTCATACTTCTCAT-3′. The PCR amplification was carried out in a 20 *μ*l final reaction mixture containing a diluted cDNA solution, 10 *μ*M of each primer and 10 *μ*l SYBR-Green PCR Master Mix. The thermal cycling conditions were presented as the following: one cycle at 95°C for 10 min, 40 cycles at 95°C for 15 sec and then one cycle at 60°C for 60 sec. The data were analyzed using the 2^−ΔΔCT^ method as previously described (22).

### Western blot analysis for HIF-1α and MDR1/P-gp

The Hep-2 cells were harvested and lysed with a cold radio-immunoprecipitation assay (RIPA) protein lysis buffer. Following sonication and incubation on ice for 30 min, the lysates were transferred into Eppendorf tubes, which were centrifugated at 12,000 × g for 40 min at 4°C. The protein concentration of the supernatant was determined by the Bradford method. The samples were then boiled at 95°C for 5 min and loaded onto SDS-PAGE (5% stacking gel and 8% separating gel for HIF-1α and MDR1/P-gp), followed by a separation at 80 V for ∼2 h, prior to being transferred onto a polyvinylidene difluoride (PVDF) membrane (Millipore). The membrane was blocked using 4% skimmed milk for 1.5 h at room temperature. Following this, the samples were incubated with primary antibodies overnight at 4°C (HIF-1α 1:100, mouse anti-human; MDR1/P-pg, 1:200, mouse anti-human; and β-actin, 1:1,000, rabbit anti-human). Horseradish peroxidase-conjugated secondary antibodies against rabbit or mouse IgG (BossBio, Beijing, China) were used to incubate the membrane for 1 h at room temperature. Finally, the protein gel bands were detected by enhanced chemiluminescence (ECL; Amersham Pharma Biotech, Amersham, UK), according to the manufacturer’s instructions.

### Statistical analysis

Statistical analysis was performed using SPSS 13.0 statistical software (Chicago, IL, USA). The categorical variables were assessed by χ^2^ or Fisher’s exact tests. Comparisons of the quantitative variables were analyzed by the Student’s t-test or a one-way ANOVA. The Spearman’s rank correlation test was used to determine the correlation between HIF-1α and MDR1/P-pg expression. For all tests, P<0.05 was considered to indicate a statistically significant difference.

## Results

### Expression of HIF-1α and MDR1/P-gp in human LSCC tissue

**Figure 1.**
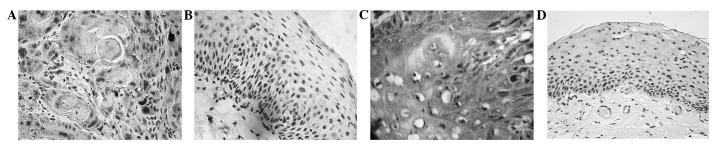
Immunohistochemical staining of HIF-1α and MDR1/P-gp in human laryngeal carcinoma and normal laryngeal epithelium. (A) HIF-1α was expressed in the nuclei and cytoplasm of the tumor cells (×400). (B) HIF-1α expression was negative in the normal laryngeal epithelium (×400). (C) MDR1/P-gp was mainly localized in the cytoplasm and cytomembrane of the tumor cells (×400). (D) MDR1/P-gp expression was negative in the normal laryngeal epithelium (×400). HIF-1α, hypoxia-inducible factor-1α; MDR1/P-gp, MDR1/P-glycoprotein.

Immunostaining for HIF-1α was observed in 56 (65.1%) of the 86 laryngeal cancer tissues. The staining was not only localized in the cell nuclei, but was also observed in the cytoplasm of the tumor cells (Fig. 1A). However, HIF-1α nuclear staining was negatively expressed in the 32 normal laryngeal mucosa tissues (P<0.05; Fig. 1B). MDR1/P-gp immunostaining was identified in 34 (39.5%) of the 86 LSCC tissue samples and was predominantly expressed in the cytoplasm and cytomembrane of the LSCC cells (Fig. 1C). In contrast, MDR1/P-gp expression was only observed in 3.1% (1/32) of the normal laryngeal mucosa samples (Fig. 1D), which was significantly lower than in the laryngeal cancer samples (P<0.05). Among the LSCC samples, HIF-1α expression was closely associated with the clinical stage and level of lymphatic invasion of the tumor cells (P<0.05). However, no differences were noted in the HIF-1α expression following changes in the age of the patients or the histological grade or primary location of the tumors (P>0.05; Table I). MDR1/P-pg expression in the human laryngeal tissue was not correlated with age or primary site (P>0.05), but was correlated with the clinical stage, differentiation grade and lymph node metastasis (P<0.05; Table I). Furthermore, a positive linear correlation was observed between HIF-1α and MDR1/P-pg expression in the LSCC tissues (r=0.442, P<0.01; Table II).

### Hypoxia induces HIF-1α and MDR1 expression

**Figure 2.**
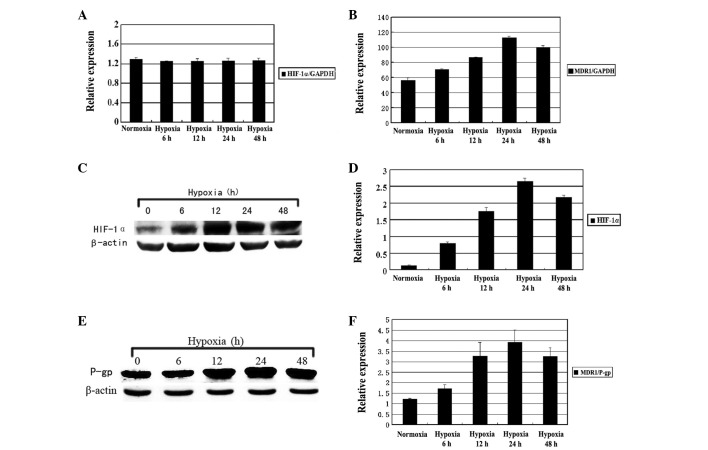
Expression of *HIF-1α* and the *MDR1* gene in Hep-2 cells under normoxic and hypoxic conditions. Hep-2 cells were incubated in 10% fetal bovine serum medium under normoxia or hypoxia at the indicated times. The relative values of (A) *HIF-1α* and (B) *MDR1* mRNA to *GAPDH* mRNA were detected by QRT-PCR. The expression of (C) HIF-1α and (D) MDR1/P-gp in the Hep-2 cells was determined by western blot analysis. The graphs represent the gray values (relative expression of the protein) of (E) HIF-1α and (F) MDR1/P-gp. N, normoxia; H, hypoxia; HIF-1α, hypoxia-inducible factor-1α; MDR1/P-gp, MDR1/P-glycoprotein; QRT-PCR, real-time quantitative reverse transcription PCR.

**Figure 3.**
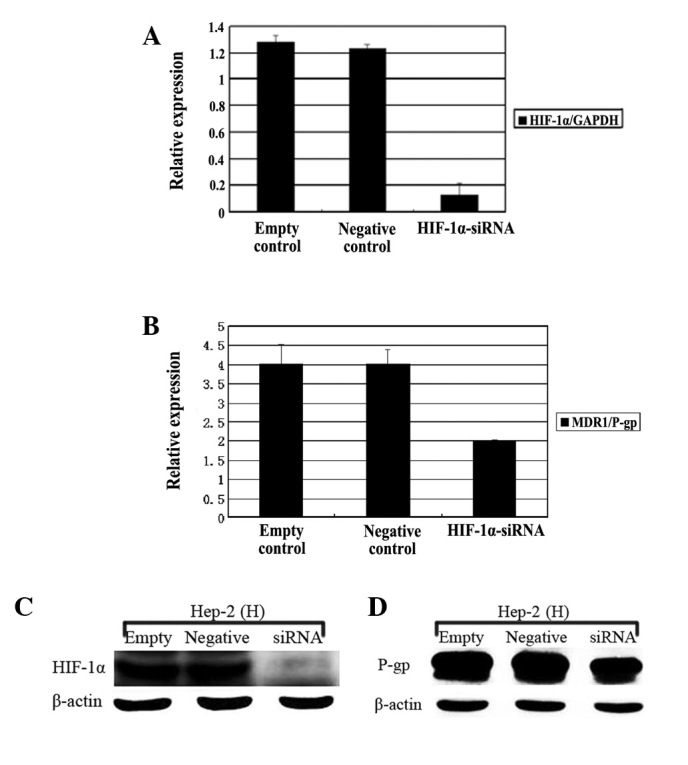
Downregulated HIF-1α expression repressing the expression of the *MDR1* gene in hypoxic Hep-2 cells. Hep-2 cells were transfected with either a vector containing a *HIF-1α* scrambled or a vector containing a *HIF-1α* targeting sequence (HIF-1α-siRNA) and then incubated in hypoxic conditions for 24 h. Total RNA was then isolated and analyzed by QRT-PCR for (A) *HIF-1α* and (B) *MDR1* mRNA expression. Cell lysates were prepared and detected by western blot analysis for (C) HIF-1α and (D) MDR1/P-gp expression. HIF-1α, hypoxia-inducible factor-1α; MDR1/P-gp, MDR1/P-glycoprotein; QRT-PCR, real-time quantitative reverse transcription PCR.

When the Hep-2 cells were exposed to hypoxia for 6, 12, 24 or 48 h, the expression of *HIF-1α* and *MDR1* was detected by QRT-PCR and western blot analysis. As shown in Figure 2, the *MDR1* mRNA expression was gradually elevated as the hypoxia was prolonged when compared with the normoxic group. The maximum level of *MDR1* mRNA was observed at 24 h (P<0.01). However, no significant differences were observed in the expression levels of the HIF-1α mRNA (P>0.05). Western blot analysis revealed that the hypoxia caused a time-dependent increase in the expression of the HIF-1α and MDR1/P-gp proteins, reaching a climax at 24 h under hypoxic conditions (P<0.05; Fig. 3).

### Role of HIF-1α in MDR1 gene induction

In order to explore the role of HIF-1α in hypoxia-induced *MDR1* gene expression, the Hep-2 cells were transfected with a double stranded siRNA oligonucleotide targeting the *HIF-1α* gene or non-specific control siRNA for 24 h prior to incubation under normoxic or hypoxic conditions. As shown in Figure 3A and B, in comparison with the negative or untreated controls, the mRNA and protein expression of HIF-1α in the hypoxic Hep-2 cells was markedly reduced following transfection by the siRNA targeting the *HIF-1α* gene (HIF-1α-siRNA; P<0.01). Similarly, Fig. 3C and D showed that the HIF-1α-siRNA also resulted in the significant downregulation of the *MDR1* mRNA and protein in the Hep-2 cells that were cultured under hypoxic conditions (P<0.05).

## Discussion

LSCC is one of the most common solid malignancies of the head and neck region. At present, the molecular mechanisms that contribute to the invasion, metastasis and resistance to chemotherapy by the LSCC cells remain unclear. Thus, it is of vital importance that potential biomarkers are identified that reflect the biological characteristics of neoplasms, in addition to the prognosis of patients. HIF-1α has previously been demonstrated to perform a vital role in modulating the biological characteristics of tumor cells, including angiogenesis, unlimited growth and resistance to chemotherapy (21). Currently, there is no shortage of controversy with regard to a correlation between HIF-1α expression and the tumor progression and lymph node metastasis of LSCC. Wu *et al* (11) indicated that HIF-1α expression in laryngeal cancer tissues was closely associated with tumor stage and lymph node metastasis. However, Cabanillas *et al* (12) observed that HIF-1α expression in LSCC cells correlated with the T-classification of tumors, but was not associated with any other clinicopathological variables. The data from the present study were consistent with the results from the study by Wu *et al*, confirming that the expression of HIF-1α in human LSCC tissues was significantly associated with the tumor stage and lymph node metastasis. The assessment of HIF-1α expression in the LSCC tissues may be conducive to predicting the status of tumor progression and metastasis.

It is well known that MDR1/P-gp is considered a bifunctional regulator of multidrug resistance that exists in a wide variety of tumors, including LSCC (14,15). Additionally, several studies have revealed that MDR1/P-pg may have an effect on promoting the invasion of human cancer cells (23,24). To date, substantial attention has been focused on the correlation between MDR1/P-gp expression and the clinicopathological features in human malignancies. Certain studies demonstrated that MDR1/P-gp expression in human cancer is associated with histological differentiation (25), tumor stages (16) and lymphatic invasion (16). However, Sagol *et al* (26) showed that no significant correlation was evident between MDR1/P-gp expression and the clinicopathological variables in pancreatic carcinoma. To further strengthen the evidence linking MDR1/ P-gp expression with the clinical status of LSCC, the present study investigated the expression level of MDR1/P-gp in 86 cases of human LSCC tissue. Accordingly, the data revealed that the MDR1/P-gp expression in the laryngeal cancer tissues was closely correlated with tumor stage, lymph node metastasis and histological grade, suggesting that MDR1/P-gp may have an effect on the tumor progression and cell differentiation of LSCC. At present, the *in vivo* study of the correlation between HIF-1α and MDR1/P-gp expression in human cancer cells remains limited. The present data provided evidence of a positive correlation between HIF-1α and MDR1/P-gp expression in human LSCC tissues.

It is commonly accepted that hypoxia is able to induce a change in the physiology and biochemistry of tumor cells by regulating the expression of multiple genes in order to adapt to a hypoxic microenvironment. Previous studies have confirmed that hypoxia may take part in the regulation of tumor cell chemoresistance (27,28). In particular, a number of studies have revealed that hypoxia may contribute to chemoresistance by enhancing the expression of the *MDR1* gene in tumor cells (27,28). However, Song *et al* (29) indicated that the regulation of *MDR1* gene expression may not be involved in hypoxia-induced chemoresistance in human non-small cell lung cancer. The present data showed that hypoxia had a significant effect on mediating the upregulation of *MDR1* gene expression in the laryngeal carcinoma Hep-2 cells. The differences between the previously cited studies may have been due to the intrinsic distinctions in the molecular mechanisms for the regulation of chemoresistance in the various types of neoplastic cells.

HIF-1α is a major transcriptional regulator of multiple target genes that are implicated in cellular adaptive responses to hypoxia (21). The present study confirmed that the HIF-1α protein was minimally expressed in the Hep-2 cells under normoxic conditions, whereas its expression was markedly increased in the hypoxic Hep-2 cells, further supporting the theory that HIF-1α is an oxygen-regulated protein. To the best of our knowledge, multiple *in vitro* studies have demonstrated that HIF-1α may play a key role in the induction of *MDR1* gene expression in tumor cells under hypoxic conditions (30,31). Unfortunately, the role of HIF-1α in the regulation of *MDR1* gene expression in laryngeal cancer cells under hypoxic conditions has not yet been clarified. In the present study, a significant reduction of *MDR1* gene expression in the hypoxic Hep-2 cells was observed following the inhibition of HIF-1α expression using transfected siRNA molecules, suggesting that HIF-1α may play an integral role in the regulation of *MDR1* gene expression in hypoxic LSCC cells. To date, the molecular mechanisms of HIF-1α-regulated *MDR1* gene expression in laryngeal cancer cells remain undefined and require further investigation.

The present study has shown that HIF-1α expression is significantly correlated with MDR1/P-gp expression in LSCC, and that the two proteins may serve as potential biomarkers for predicting the malignant progression and metastasis of human LSCC. Moreover, the data demonstrate that hypoxia may enhance the expression of the *MDR1* gene in Hep-2 cells. As a key nuclear transcription factor, HIF-1α exerts a positive regulatory effect on *MDR1* gene expression in response to hypoxia in laryngeal cancer Hep-2 cells. Thus, targeting the HIF-1α/MDR1/P-gp signaling pathway may be a potential therapeutic strategy for treating LSCC.

## Figures and Tables

**Table I.** Correlation between clinicopathological features and HIF-1α and MDR1/P-gp expression in 86 cases of human laryngeal carcinoma.

Clinicopathological parameter	N	HIF-1 α, n		χ^2^	P-value	MDR1/P-gp, n		χ^2^	P-value
		Positive	Negative			Positive	Negative		
Age (years)				1.061	0.303			2.237	0.135
<60	55	38	17			25	30		
≥60	31	18	13			9	22		
Primary location				0.665	0.717			3.518	0.172
Supraglottic	18	13	5			8	10		
Glottic	66	42	24			24	42		
Subglottic	2	1	1			2	0		
Histological grade				0.066	0.968			6.658	0.038
I	29	19	10			6	23		
II	44	28	16			21	23		
III	13	8	5			7	6		
Clinical stage				5.346	0.021			5.978	0.024
I–II	63	36	27			20	43		
III–IV	23	20	3			14	9		
Lymph node status				4.417	0.036			4.433	0.035
Positive	18	16	2			11	7		
Negative	68	40	28			23	45		

HIF-1α, hypoxia-inducible factor-1α; MDR1/P-gp, MDR1/P-glycoprotein.

**Table II.** Correlation between HIF-1α and MDR1/P-gp expression in human laryngeal carcinoma tissue.

MDR1/P-gp (No. of cases)	HIF-1α (No. of cases)		
	Positive	Negative	Total
Positive	31	25	56
Negative	3	27	30
Total	34	52	86

R=0.442; P<0.01, HIF-1α vs. MDR1/P-gp expression. HIF-1α, hypoxia-inducible factor-1α; MDR1/P-gp, MDR1/P-glycoprotein.
